# Characterization of *Salmonella enterica* isolates causing bacteremia in Lima, Peru, using multiple typing methods

**DOI:** 10.1371/journal.pone.0189946

**Published:** 2017-12-21

**Authors:** Claudia Silva, Laura Betancor, Coralith García, Lizeth Astocondor, Noemí Hinostroza, Julieta Bisio, Javier Rivera, Lucía Perezgasga, Victoria Pérez Escanda, Lucía Yim, Jan Jacobs, Francisco García-del Portillo, the SalmoIber CYTED Network, José A. Chabalgoity, José L. Puente

**Affiliations:** 1 Departamento de Microbiología Molecular, Instituto de Biotecnología, Universidad Nacional Autónoma de México, Cuernavaca, Morelos, Mexico; 2 Instituto de Higiene, Facultad de Medicina, Universidad de la República, Montevideo, Uruguay; 3 Instituto de Medicina Tropical Alexander von Humboldt, Universidad Peruana Cayetano Heredia, Lima, Peru; 4 Programa de Ingeniería Genómica, Centro de Ciencias Genómicas, Universidad Nacional Autónoma de México, Cuernavaca, Morelos, Mexico; 5 Institute of Tropical Medicine Antwerp, Antwerp, Belgium; 6 Laboratorio of Patógenos Bacterianos Intracelulares, Centro Nacional de Biotecnología-Consejo Superior de Investigaciones Científicas (CNB-CSIC), Madrid, Spain; Panjab University, INDIA

## Abstract

In this study, different molecular typing tools were applied to characterize 95 *Salmonella enterica* blood isolates collected between 2008 and 2013 from patients at nine public hospitals in Lima, Peru. Combined results of multiplex PCR serotyping, two- and seven-loci multilocus sequence typing (MLST) schemes, serotyping, IS200 amplification and RAPD fingerprints, showed that these infections were caused by eight different serovars: Enteritidis, Typhimurium, Typhi, Choleraesuis, Dublin, Paratyphi A, Paratyphi B and Infantis. Among these, Enteritidis, Typhimurium and Typhi were the most prevalent, representing 45, 36 and 11% of the isolates, respectively. Most isolates (74%) were not resistant to ten primarily used antimicrobial drugs; however, 37% of the strains showed intermediate susceptibility to ciprofloxacin (ISC). Antimicrobial resistance integrons were carried by one Dublin (*dfra1* and *aadA1*) and two Infantis (*aadA1*) isolates. The two Infantis isolates were multidrug resistant and harbored a large megaplasmid. Amplification of *spvC* and *spvRA* regions showed that all Enteritidis (n = 42), Typhimurium (n = 34), Choleraesuis (n = 3) and Dublin (n = 1) isolates carried the *Salmonella* virulence plasmid (pSV). We conclude that the classic serotyping method can be substituted by the multiplex PCR and, when necessary, sequencing of only one or two loci of the MLST scheme is a valuable tool to confirm the results. The effectiveness and feasibility of different typing tools is discussed.

## Introduction

*Salmonella* is one of the most prevalent foodborne pathogens worldwide. Clinical manifestations of salmonellosis vary from self-limiting diarrhea to systemic disease, particularly in susceptible individuals including infants, immune-compromised patients and the elderly [1]. Severe infections with bacteremia and meningitis can be caused both by typhoid and non-typhoidal *Salmonella* [2].

*Salmonella* classification is based on the characterization of O (somatic) and H (flagellar) antigens by agglutination with hyperimmune antisera according to the Kauffmann-White-LeMinor scheme [3]. Classic serotyping has been used for decades in foodborne disease surveillance and outbreak investigations; however, serotyping requires more than 250 different typing antisera and 350 different antigens for preparation and quality control. Serotyping is laborious, time consuming and, due to production variability, it can lead to imprecise results. Molecular methods have been developed to complement or replace traditional serotyping to classify *Salmonella* isolates [4–6]. Molecular typing methods are highly sensitive, very specific, fast and show better standardization and reproducibility than traditional methods [7].

Molecular serotyping is based on the identification of unique gene sequences to determine O, H1 and H2 antigens [7–9]. Multilocus sequence typing (MLST) is based on the determination of the nucleotide sequences of internal regions of a series of housekeeping genes common to all *Salmonella enterica* serovars [10]. MLST is highly reproducible and results can be easily exchanged between laboratories via an online public *Salmonella enterica* database (mlst.warwick.ac.uk/mlst/dbs/Senterica/) [4]. Indeed, MLST has been proposed as an alternative system for classification of *Salmonella* isolates replacing traditional serotyping [4, 6].

Epidemiology studies indicate that molecular tools are needed to analyze the genetic variation within a serovar. For this purpose, the most used subtyping technique is the macro-restriction profile generated by rarely-cutting restriction enzymes, which is resolved by pulse field gel electrophoresis (PFGE) [11]; however, PGFE is labor-intensive, time-consuming and requires specialized equipment. An alternative practical methodology is random amplified polymorphic DNA (RAPD) analysis [12]. The advantages and disadvantages of the *Salmonella* molecular typing methods have previously been discussed in detail [5, 6, 13].

Other methods allow the identification of specific serovars by amplification of a unique sequence. For example, *S*. *enterica* serovar Typhi strains possess a distinctive copy of the IS*200* insertion element between the *gyrA* and *rcsC* genes [14], a particular feature that could be used for taxonomy and epidemiology [15].

Many of the features involved in *Salmonella* virulence and antimicrobial resistance are encoded by genes acquired by horizontal transfer events and thus, present only in particular strains as part of their “accessory genome” (*e*.*g*. plasmids, transposons and integrons) [16]. Eight *S*. *enterica* serovars harbour a large (50–285 kb) plasmid named the *Salmonella* virulence plasmid (pSV) containing the *spv* operon, which is a major determinant of virulence in their specific hosts [17–21]. pSV is not required to cause gastroenteritis and its association with human bacteremia is still in debate [18, 22, 23]. Several studies have addressed the increased risk for hospitalization, invasive illness, and death that represent multidrug resistant (MDR) *Salmonella* [24, 25]. MDR is frequently associated with plasmids carrying multiple resistance determinants in transposons and integrons [26–29].

Limited information on current *Salmonella* epidemiology is available in Peru [30], particularly regarding the serotypes causing bacteremia. In this study, we analyzed a collection of isolates causing bacteremia in patients attending nine public hospitals in Lima, Peru. Different approaches, including multiplex PCR serotyping, two- and seven-loci MLST, antimicrobial susceptibility tests, RAPD fingerprints and PCR probing for the presence of multidrug resistance-associated integrons, the pSV virulence plasmid and the *gyrA*-*rcsC* IS200 insertion, were used to determine the serovar and explore the diversity of the isolates. We discuss the advantages and disadvantages of each method in the context of the epidemiological setting in developing countries.

## Materials and methods

### Ethics statement

Blood samples were collected by laboratory technicians as part of the patients’ diagnostic tests in each of the nine hospitals included in this report. Microbiologists carrying out the *Salmonella* identification omitted any disclosure of the patient’s identity. *Salmonella* isolates were characterized in research laboratories without access to patient information. This collection was part of a surveillance study focused on antimicrobial resistance among key blood isolates pathogens that involved nine hospitals of Lima during the study period. Blood cultures were not taken as part of the study procedures. For instance, written or verbal consent were not obtained since blood cultures were performed as part of routine clinical care. The study procedures including the omission of need of written/oral consent were approved by the Institutional Review Board of the Universidad Peruana Cayetano Heredia and by each hospital.

### Bacterial strains

Bacterial isolates were collected as part of a microbiological surveillance study from 2008 to 2013 through a network of nine public hospitals in Lima, Peru (S1 Fig). Blood cultures were drawn during routine patient care. The isolates identified as *Salmonella* were gather together at the Instituto de Medicina Tropical Alexander von Humboldt and stored at -70° C. The isolates were cultured in MacConkey agar, and since the present study was intended to analyze non-typhoidal *Salmonella*, those lactose-negative isolates with typical biochemical characteristics of Typhi were not included. In the present study a total of 95 isolates were analyzed.

### Antimicrobial susceptibility testing

Antimicrobial resistance of isolates SPE1 to SPE100 was tested by the Kirby–Bauer disk diffusion method [31], using the following antibiotic disks (Oxoid™): trimethoprim-sulfamethoxazole (25 μg), tetracycline (30 μg), nalidixic acid (30 μg), ampicillin (10 μg), cefotaxime (30 μg), ceftazidime (30 μg), aztreonam (30 μg), ceftriaxone (30 μg), chloramphenicol (30 μg) and ciprofloxacin (5 μg). The breakpoints were considered according to the Clinical Laboratory Standards Institute [32].

### DNA extraction

For the serovar determination by multiplex-PCR, the DNA was extracted by boiling a single culture colony of each isolate, as described elsewhere [12]. For the other PCR procedures DNA was extracted from liquid cultures by a modification of the salt extraction method [33]. Detailed protocols are described in S1 Text.

### Determination of specific *Salmonella* O somatic and flagellar antigens by multiplex PCR

To determine the *Salmonella* O somatic antigen, primers pairs *wzxB*, *wzxC1*, *wzx-C2*, *wzx-E* and *tyvD* (S1 Table), previously reported by Herrera-León *et al*. (2007) [7], corresponding to the *wzx* gene from serogroups B (O:4), C1 (O:7), C2 (O:8) and E (O:3) and the *tyv* gene from serogroup D (O:9), respectively, were used. Amplification was performed in 20 μl reactions containing 1 U of Taq polymerase, 3mM MgCl, 0.2 mM dNTPs and 0.2 μM of each primer pair, using a commercial Taq polymerase kit (Thermo scientific). Two μL of colony extracted total DNA was used as template. The cycling program was as follows: 5 min 95°C followed by 30 cycles of 40 s at 94°C, 30 s at 58°C and 30 s at 72°C, and completed by a final extension for 5 minutes at 72°C. The amplification products were separated in 2.5% agarose gels in 0.5X TBE buffer. Fragment size was determined by comparing with a 100 bp ladder (Thermo) and with the products obtained using control DNAs for serovars Typhimurium, Livingstone, Hadar, Enteritidis and Anatum to address for serogroups B (O:4), C1 (O:7), C2 (O:8), D (O:9) and E (O:3), respectively.

First and second phase flagellar antigens were determined using the multiplex PCR previously reported [8, 9]. The PCR reaction to determine first phase flagellar type is targeted to the *fliC* gene and uses 1 forward primer (Sense 60) and 6 reverse primers specific for i, z-10, b, eh, lv and r flagellar types, plus specific primer pairs for d type and G complex flagellar type. A primer pair specific for the *sdf* gene was included in order to identify Enteritidis among the G complex serovars (S1 Table).

Amplification was performed in 20 μl reactions as described above using 0.2 μM each of primers i, z, lv, r and *sdf*, and 0.3 μM each of primers b, eh, d and G. Colony extracted total DNA (2 μL) was used as template. The cycling program was the same as for serogroup PCR. Fragment size was determined by comparing with a 100 bp ladder (Thermo) and with the products obtained using control DNAs for serovars Typhi, Paratyphi B, Anatum, Typhimurium, Infantis, Panama, Hadar and Enteritidis to address for flagellar antigens d, b, eh, i, r, lv, z10 and G, respectively.

The PCR reaction to determine second phase flagellar antigen is targeted to the *fljB* gene and uses 10 primers to identify 7 different flagellar types (1,2; 1,5; 1,6; 1,7; l,w; e,n,x and e,n,z15) (S1 Table). The amplification and visualization of products were performed in identical conditions than for *fliC* and using control DNAs for serovars Typhimurium, Infantis, Anatum, Bredeney, Livingstone, Branderup and Hadar to determine 1,2; 1,5; 1,6; 1,7; l,w; e,n,z15 and e,n,x, respectively.

### Serology

The serology was performed according to the Kauffmann-White-LeMinor scheme and the protocol of Grimont and Weill (2007) (https://www.pasteur.fr/sites/default/files/veng_0.pdf), based on the slide agglutination test using a combination of commercial (Prolab) and custom prepared anti O and anti H sera.

### Multilocus sequence typing (MLST)

The complete seven-loci MLST was applied for representative isolates based on the partial sequences of the following seven housekeeping genes: *aroC*, *dnaN*, *hemD*, *hisD*, *purE*, *sucA* and *thrA*. The primers for PCR and sequencing (S1 Table) were previously described by Kidgell *et al*. (2002) [34]. In addition, a *hemD-purE* two-loci analysis of the complete collection of blood isolates was performed (see results). PCR amplifications were performed as described above but in 50 μl reactions containing 0.5 μM of each primer plus 2 μL of extracted total DNA (roughly 50 ng) as a template. The cycling program was as follows: 5 min 95°C followed by 30 cycles of 30 s at 94°C, 30 s at 55°C and 45 s at 72°C and completed by a final extension for 5 minutes at 72°C. The amplification products were separated in 1% agarose gels in 0.5X TAE buffer. The products were purified with a PCR purification kit from Qiagen (Valencia, California, USA) according to the manufacturer’s recommendation, and submitted for sequencing at Macrogen (Seoul, South Korea).

Sequences were edited and aligned using Clustal W as implemented in BioEdit [35], and submitted to the *Salmonella* MLST database for allele number assignment and, in the case of the complete seven-loci scheme, for sequence type (ST) assignation (http://mlst.warwick.ac.uk/mlst/dbs/Senterica). The data introduced in the MLST database is automatically included in the Enterobase (http://enterobase.warwick.ac.uk/species/index/senterica), which is a much larger database which contains the data for the MLST database plus STs retrieved from reported *Salmonella* complete genomes. In addition, in EnteroBase the STs are arranged into eBURST clonal complex groups, which define a founder and derived genotypes [4, 36]. The alleles and STs obtained for Peruvian isolates were compared with those reported in the EnteroBase.

### PCR identification of specific traits

To confirm the classification of some isolates as serovar Typhi, the *gyrA-*IS200-*rcsC* PCR method that discriminates between Typhi and other serovars [14] was used as described by Martinez-Gamboa *et al*. (2015) [15]. Primers for *spvC* and for *spvRA* were used to determine the presence of the *Salmonella* virulence plasmid (pSV) [37]. To establish the identity of the plasmid found in the Infantis isolates, the *traC* primers were used to amplify a marker of the megaplasmid pESI, previously described for this serovar [38]. For the detection of integrons the conserved sequence (CS) primers were used [39]. All the primers used in this study are listed in S1 Table. Amplifications were performed in 50 μl reactions using commercial Taq polymerase kit (Thermo scientific), using 1.5 U Taq polymerase per tube, and with a final concentration of 1.5 mM MgCl, 0.2 mM dNTPs and 0.5 μM of each primer. Extracted total DNA (2 μL) was used as a template (roughly 50 ng). The cycling program was as follows: 5 min 95°C followed by 30 cycles of 45 s at 94°C, 30 s at 55°C and 45 s at 72°C and completed by a final extension for 5 minutes at 72°C. The amplification products were separated in 1% agarose gels in 0.5X TAE buffer. Fragment size was determined by comparison with a 100 bp ladder (Thermo). To sequence integron PCR products the same primers were used (S1 Table). The products were purified with a PCR purification kit from Qiagen (Valencia, California, USA) according to the manufacturer’s recommendation, and submitted for sequencing at Macrogen (Seoul, South Korea). Sequences were edited and aligned using Clustal W as implemented in BioEdit (Hall, 1999), and compared with sequences available in the GeneBank using BLASTn (https://blast.ncbi.nlm.nih.gov/Blast.cgi).

### Plasmid profiling

To analyze the plasmid content for selected isolates, a modified protocol of the alkaline lysis procedure proposed by Kieser [40] was used (S1 Text). The products were separated in 0.7% agarose gels in 1 X TBE buffer at 100 volts for 5 hours and stained with a 1% ethidium bromide solution and photographed.

### Random amplified polymorphic DNA (RAPD) PCR

Initially, amplifications were performed using primers OPB-15, OPB-17 and P1254 (S1 Table), previously described by Lin *et al*. (1996) [12]. Due to the lack of variability shown by the OPB-17 and P1254 primers (data not shown), only OPB-15 was used to analyze the complete *Salmonella* collection. The amplification reaction was performed in 40 μl containing 1.5 U of Taq polymerase, 3 mM MgCl, 0.2 mM dNTPs, 2 μM of primer and, as template, 2 μL (roughly 50 ng) of extracted total DNA of each strain, using a commercial Taq polymerase kit (Thermo scientific). The cycling program was as follows: 3 min at 95°C, 3 min at 35°C and 3 min 30 s at 72°C for 3 cycles, followed by 25 cycles of 30 s at 94°C, 1 min at 35°C and 1 min 30 s at 72°C, and completed by a final extension for 5 minutes at 72°C. The amplification products were resolved in 1% agarose gels in 0.5X TAE buffer. Fragment size was determined by comparing with a 100 bp ladder (Thermo).

## Results

### PCR amplification of somatic, flagellar and IS200 specific sequences successfully predicted the *Salmonella* serovars of most bloodstream isolates

The 95 blood isolates collected during 2008 to 2013 from nine public hospitals in Lima, Peru, which were classified as *Salmonella* spp. by biochemical methods, were subjected, as described in Materials and Methods, to multiplex PCR [7–9] to determine their serovar (S2 Fig). Most of the isolates (94%) were successfully typed and assigned to a known serovar; only six isolates remained as undetermined because rendered no amplification product in one of the three PCR reactions. The majority of the isolates were unambiguously determined to be serovars Enteritidis (n = 42), Typhimurium (n = 34), Typhi (n = 10), Infantis (n = 2) and Paratyphi B (n = 1) (Table 1).

**Table 1:** Characterization of *Salmonella enterica* isolates causing bacteremia in Lima, Peru.

Strain	Hospital ^a^	Date	Resistance profile ^b^	PCR-predicted O and H type	PCR-predicted serovar	Antigenic formula	Serology-predicted serovar	RAPD	*spvC* and *spvRA* ^c^	*hemD* ^d^	*purE* ^d^	Sequence-predicted serovar ^d^
SPE18	CH	Mar-08	NR	O:9: G:—sdf+	Enteritidis	ND	ND	A	+	*3*	*6*	Enteritidis
SPE6	AS	Apr-08	NR^-CIP^	O:9: G:—sdf+	Enteritidis	ND	ND	A	+	*3*	*6*	Enteritidis
SPE85	GA	May-08	NR	O:9: G:—sdf+	Enteritidis	ND	ND	A	+	*3*	*6*	Enteritidis
SPE58	ER	Aug-08	NR^-CIP^	O:9: G:—sdf+	Enteritidis	ND	ND	A	+	*3*	*6*	Enteritidis
SPE59	ER	Oct-08	NR^-CIP^	O:9: G:—sdf+	Enteritidis	ND	ND	A	+	*3*	*6*	Enteritidis
SPE72	GA	Dec-08	NR	O:9: G:—sdf+	Enteritidis	ND	ND	A	+	*3*	*6*	Enteritidis
SPE67	GA	Dec-08	NR^-CIP^	O:9: G:—sdf+	Enteritidis	ND	ND	A	+	*3*	*6*	Enteritidis
SPE78	GA	Dec-08	NR	O:9: G:—sdf+	Enteritidis	9: gm: -	Enteritidis	A	+	*3*	*6*	Enteritidis
SPE60	ER	Jan-09	NR	O:9: G:—sdf+	Enteritidis	ND	ND	A	+	*3*	*6*	Enteritidis
SPE62	ER	Feb-09	NR^-CIP^	O:9: G:—sdf+	Enteritidis	ND	ND	A	+	*3*	*6*	Enteritidis
SPE66	GA	Mar-09	NR	O:9: G:—sdf+	Enteritidis	ND	ND	A	+	*3*	*6*	Enteritidis
SPE77	GA	Apr-09	NR	O:9: G:—sdf+	Enteritidis	ND	ND	A	+	*3*	*6*	Enteritidis
SPE98	SB	Jun-09	NR	O:9: G:—sdf+	Enteritidis	ND	ND	A	+	*3*	*6*	Enteritidis
SPE64	ER	Jun-09	NAL^-CIP^	O:9: G:—sdf+	Enteritidis	ND	ND	A	+	*3*	*6*	Enteritidis
SPE4	AL	Aug-09	TET	O:9: G:—sdf+	Enteritidis	ND	ND	A	+	*3*	*6*	Enteritidis
SPE5	AL	Aug-09	NR	O:9: G:—sdf+	Enteritidis	ND	ND	A	+	*3*	*6*	Enteritidis
SPE83	GA	Nov-09	NR	O:9: G:—sdf+	Enteritidis	ND	ND	A	+	*3*	*6*	Enteritidis
SPE71	GA	Dec-09	NR	O:9: G:—sdf+	Enteritidis	ND	ND	A	+	*3*	*6*	Enteritidis
SPE84	GA	Jan-10	NAL^-CIP^	O:9: G:—sdf+	Enteritidis	ND	ND	A	+	*3*	*6*	Enteritidis
SPE69	GA	Feb-10	NR	O:9: G:—sdf+	Enteritidis	ND	ND	A	+	*3*	*6*	Enteritidis
SPE24	CH	Feb-10	NR	O:9: G:—sdf+	Enteritidis	ND	ND	A	+	*3*	*6*	Enteritidis
SPE82	GA	Feb-10	NAL^CIP^	O:9: G:—sdf+	Enteritidis	ND	ND	A	+	*3*	*6*	Enteritidis
SPE86	GA	Feb-10	NR	O:9: G:—sdf+	Enteritidis	9: gm: -	Enteritidis	A	+	*3*	*6*	Enteritidis
SPE51	DA	Mar-10	NR	O:9: G:—sdf+	Enteritidis	ND	ND	A	+	*3*	*6*	Enteritidis
SPE80	GA	Mar-10	NR^-CIP^	O:9: G:—sdf+	Enteritidis	ND	ND	A	+	*3*	*6*	Enteritidis
SPE52	DA	Mar-10	NR	O:9: G:—sdf+	Enteritidis	ND	ND	A	+	*3*	*6*	Enteritidis
SPE53	DA	Mar-10	NR	O:9: G:—sdf+	Enteritidis	ND	ND	A	+	*3*	*6*	Enteritidis
SPE90	HU	Mar-10	NR^-CIP^	O:9: G:—sdf+	Enteritidis	ND	ND	A	+	*3*	*6*	Enteritidis
SPE14	AS	Apr-10	NR^-CIP^	O:9: G:—sdf+	Enteritidis	ND	ND	A	+	*3*	*6*	Enteritidis
SPE32	CH	Apr-10	NR	O:9: G:—sdf+	Enteritidis	ND	ND	A	+	*3*	*6*	Enteritidis
SPE81	GA	Apr-10	NR	O:9: G:—sdf+	Enteritidis	ND	ND	A	+	*3*	*6*	Enteritidis
SPE16	AS	May-10	NR	O:9: G:—sdf+	Enteritidis	ND	ND	A	+	*3*	*6*	Enteritidis
SPE92	HU	Jul-10	NR^-CIP^	O:9: G:—sdf+	Enteritidis	ND	ND	A	+	*3*	*6*	Enteritidis
SPE22	CH	Aug-10	NAL^-CIP^	O:9: G:—sdf+	Enteritidis	ND	ND	A	+	*3*	*6*	Enteritidis
SPE21	CH	Aug-10	NR	O:9: G:—sdf+	Enteritidis	ND	ND	A	+	*3*	*6*	Enteritidis
SPE54	DA	Sep-10	NR	O:9: G:—sdf+	Enteritidis	ND	ND	A	+	*3*	*6*	Enteritidis
SPE93	HU	Nov-10	NR^-CIP^	O:9: G:—sdf+	Enteritidis	9: gm: -	Enteritidis	A	+	*3*	*6*	Enteritidis
SPE97	MA	May-11	NR	O:9: G:—sdf+	Enteritidis	9: gm: -	Enteritidis	A	+	*3*	*6*	Enteritidis
SPE40	CH	Dec-11	NR	O:9: G:—sdf+	Enteritidis	ND	ND	A	+	*3*	*6*	Enteritidis
SPE39	CH	Apr-12	AMP-CHL-ATM-CRO-CAZ-CTX	O:9: G:—sdf+	Enteritidis	ND	ND	A	+	*3*	*6*	Enteritidis
SPE23	CH	Aug-12	NR^-CIP^	O:9: G:—sdf+	Enteritidis	ND	ND	A	+	*3*	*6*	Enteritidis
SPE36	CH	Aug-12	NR^-CIP^	O:9: G:—sdf+	Enteritidis	ND	ND	A	+	*3*	*6*	Enteritidis
SPE7	AS	Jun-08	NR	O:4:i: 1,2	Typhimurium	4: i: 1,2	Typhimurium	B	+	*12*	*5*	**Typhimurium**
SPE47	DA	Aug-08	NR^-CIP^	O:4:i: 1,2	Typhimurium	ND	ND	B	+	*12*	*5*	**Typhimurium**
SPE19	CH	Aug-08	NR	O:4:i: 1,2	Typhimurium	ND	ND	B	+	*12*	*5*	**Typhimurium**
SPE41	CH	Oct-08	NR	O:4:i: 1,2	Typhimurium	ND	ND	C	+	*12*	*5*	**Typhimurium**
SPE8	AS	Nov-08	NR	O:4:i: 1,2	Typhimurium	ND	ND	B	+	*12*	*5*	**Typhimurium**
SPE74	GA	Nov-08	NR	O:4:i: 1,2	Typhimurium	ND	ND	C	+	*12*	*5*	**Typhimurium**
SPE9	AS	Jan-09	NR	O:4:i: 1,2	Typhimurium	ND	ND	B	+	*12*	*5*	**Typhimurium**
SPE3	AL	Feb-09	NR^-CIP^	O:4:i: 1,2	Typhimurium	4: i: 1,2	Typhimurium	C	+	*12*	*5*	**Typhimurium**
SPE87	HU	Feb-09	NR	O:4:i: 1,2	Typhimurium	ND	ND	D	+	*12*	*5*	**Typhimurium**
SPE49	DA	May-09	NR^-CIP^	O:4:i: 1,2	Typhimurium	ND	ND	B	+	*12*	*5*	**Typhimurium**
SPE11	AS	May-09	NR	O:4:i: 1,2	Typhimurium	4: i: 1,2	Typhimurium	B	+	*12*	*5*	**Typhimurium**
SPE63	ER	Jun-09	NR	O:4:i: 1,2	Typhimurium	4:i: 1,2	Typhimurium	B	+	*12*	*5*	**Typhimurium**
SPE33	CH	Jun-09	SXT-TET	O:4:i: 1,2	Typhimurium	4:i: 1,2	Typhimurium	B	+	*12*	*5*	**Typhimurium**
SPE76	GA	Aug-09	NAL-CIP	O:4:i: 1,2	Typhimurium	ND	ND	D	+	*12*	*5*	**Typhimurium**
SPE65	ER	Sep-09	NAL^-CIP^	O:4:i: 1,2	Typhimurium	ND	ND	B	+	*12*	*5*	**Typhimurium**
SPE1	AL	Jan-10	NR	O:4:i: 1,2	Typhimurium	4: i: 1,2	Typhimurium	B	+	*12*	*5*	**Typhimurium**
SPE13	AS	Feb-10	NR	O:4:i: 1,2	Typhimurium	ND	ND	B	+	*12*	*5*	**Typhimurium**
SPE96	MA	Mar-10	NR	O:4:i: 1,2	Typhimurium	ND	ND	B	+	*12*	*5*	**Typhimurium**
SPE99	SB	Mar-10	NR	O:4:i: 1,2	Typhimurium	ND	ND	B	+	*12*	*5*	**Typhimurium**
SPE50	DA	Mar-10	AMP-TET	O:4:i: 1,2	Typhimurium	ND	ND	B	+	*12*	*5*	**Typhimurium**
SPE43	CH	Mar-10	TET	O:4:i: 1,2	Typhimurium	ND	ND	B	+	*12*	*5*	**Typhimurium**
SPE79	GA	Apr-10	NR	O:4:i: 1,2	Typhimurium	ND	ND	B	+	*12*	*5*	**Typhimurium**
SPE15	AS	May-10	NR	O:4:i: 1,2	Typhimurium	ND	ND	B	+	*12*	*5*	**Typhimurium**
SPE27	CH	May-10	NR^-CIP^	O:4:i: 1,2	Typhimurium	4:i: 1,2	Typhimurium	C	+	*12*	*5*	**Typhimurium**
SPE26	CH	Jun-10	NR	O:4:i: 1,2	Typhimurium	4:i: 1,2	Typhimurium	B	+	*12*	*5*	**Typhimurium**
SPE91	HU	Jul-10	NR^-CIP^	O:4:i: 1,2	Typhimurium	ND	ND	B	+	*12*	*5*	**Typhimurium**
SPE44	CH	Aug-10	NR^-CIP^	O:4:i: 1,2	Typhimurium	4:i: 1,2	Typhimurium	B	+	*12*	*5*	**Typhimurium**
SPE17	CH	Sep-10	NR	O:4:i: 1,2	Typhimurium	ND	ND	B	+	*12*	*5*	**Typhimurium**
SPE29	CH	Nov-10	NR^-CIP^	O:4:i: 1,2	Typhimurium	ND	ND	D	+	*12*	*5*	**Typhimurium**
SPE35	CH	Dec-10	NR	O:4:i: 1,2	Typhimurium	ND	ND	B	+	*12*	*5*	**Typhimurium**
SPE37	CH	Jan-11	NR	O:4:i: 1,2	Typhimurium	ND	ND	B	+	*12*	*5*	**Typhimurium**
SPE28	CH	Jun-11	NR	O:4:i: 1,2	Typhimurium	ND	ND	B	+	*12*	*5*	**Typhimurium**
SPE31	CH	Jul-11	NR	O:4:i: 1,2	Typhimurium	4:i: 1,2	Typhimurium	B	+	*12*	*5*	**Typhimurium**
SPE42	CH	Dec-11	NR^-CIP^	O:4:i: 1,2	Typhimurium	4:i: 1,2	Typhimurium	B	+	*12*	*5*	**Typhimurium**
SPE94	MA	Jun-08	NR	O:9: d:-	Typhi	ND	ND	E	-	***2***	***1***	**Typhi**
SPE95	MA	Aug-08	NR	O:9: d:-	Typhi	ND	ND	E	-	***2***	***1***	**Typhi**
SPE73	GA	Oct-08	NAL	O:9: d:-	Typhi	9:d:-	Typhi	E	-	***2***	***1***	**Typhi**
SPE75	GA	Oct-08	NAL	O:9: d:-	Typhi	9:d:-	Typhi	E	-	***2***	***1***	**Typhi**
SPE56	ER	Dec-08	NR^-CIP^	O:9: d:-	Typhi	9:d:-	Typhi	E	-	***2***	***1***	**Typhi**
SPE61	ER	Feb-09	NR^-CIP^	O:9: d:-	Typhi	9:d:-	Typhi	E	-	***2***	***1***	**Typhi**
SPE10	AS	Feb-09	NR^-CIP^	O:9: d:-	Typhi	9: d: -	Typhi	E	-	***2***	***1***	**Typhi**
SPE30	CH	Nov-09	NR^-CIP^	O:9: d:-	Typhi	9: d: -	Typhi	E	-	***2***	***1***	**Typhi**
SPE88	HU	Jan-10	NR	O:9: d:-	Typhi	ND	ND	F	-	***2***	***1***	**Typhi**
SPE89	HU	Mar-10	NAL^-CIP^	O:9: d:-	Typhi	ND	ND	E	-	***2***	***1***	**Typhi**
SPE45	DA	May-08	NAL^-CIP^	O:7:-: 1,5	Undetermined	6,7: -: 1,5	Undetermined	G	+	*35*	*26*	Choleraesuis var Kunzendorf
SPE68	GA	Jul-08	NAL	O:7:-: 1,5	Undetermined	6,7: c: 1,5	Choleraesuis	G	+	*35*	*26*	Choleraesuis var Kunzendorf
SPE20	CH	Jan-11	NAL^-CIP^	O:7:-: 1,5	Undetermined	6,7:c: 1,5	Choleraesuis	G	+	*35*	*26*	Choleraesuis var Kunzendorf
SPE34	CH	Feb-11	NR	O:7:-: 1,5	Undetermined	6,7:c:1,5	Choleraesuis	G	+	*35*	*26*	Choleraesuis var Kunzendorf
SPE2	AL	Jan-09	SXT-TET	O:9: G:—sdf-	Undetermined	9: g, p; -	Dublin	I	+	*3*	*5*	Dublin
SPE25	CH	Apr-10	NR^-CIP^	O:9: -: 1,5	Undetermined	2: a:-	Paratyphi A	H	-	*8*	***27***	**Paratyphi A**
SPE70	GA	Aug-08	NR	O:4: b: 1,2	Paratyphi B	4: b: -	Undetermined	J	-	***24***	***37***	**Paratyphi B**
SPE55	DA	Feb-11	NAL-TET-SXT^-CIP^	O:7: r: 1,5	Infantis	6,7: r: 1,5	Infantis	K	-	***22***	*5*	**Infantis**
SPE100	CH	Jun-13	NAL-AMP-TET-SXT-CHL-ATM-CRO-CAZ-CTX^-CIP^	O:7: r: 1,5	Infantis	9: r: 1,5	Infantis	K	-	***22***	*5*	**Infantis**

NA, not available; ND, not determined.

^a^ The abbreviations for the hospitals’ names and, in parenthesis, the number of corresponding isolates are as follows: CH, Cayetano Heredia (n = 28); GA, Guillermo Almenara (n = 21); DA, Daniel Alcides Carrión (n = 9); AS, Alberto Sabogal Sologuren (n = 10); ER, Eduardo Rebagliátegui Martins (n = 9); HU, Hipólito Unanue (n = 7); AL, Arzobizpo Loayza (n = 5); MA, María Auxiliadora (n = 4); SB, Sergio Bernales (n = 2).

^b^ Abbreviations for antibiotics are: STX, trimethoprim-sulfamethoxazole; TET, tetracycline; NAL, nalidixic acid; AMP, ampicillin; CTX, cefotaxime; CAZ, ceftazidime; ATM, aztreonam; CRO, ceftriaxone; CHL, chloramphenicol; CIP, ciprofloxacin; FEP, cefepime; AMC, amoxicillin-clavulanic acid; and CXM, cefuroxime. CIP superscript indicates intermediate resistance.

^c^ +, positive amplification for at least one of the *spv* regions; -, negative amplification.

^d^ The alleles and serovars unequivocally ascribed based on comparison with the complete EnteroBase MLST database are highlighted in boldface.

**Fig 1:**
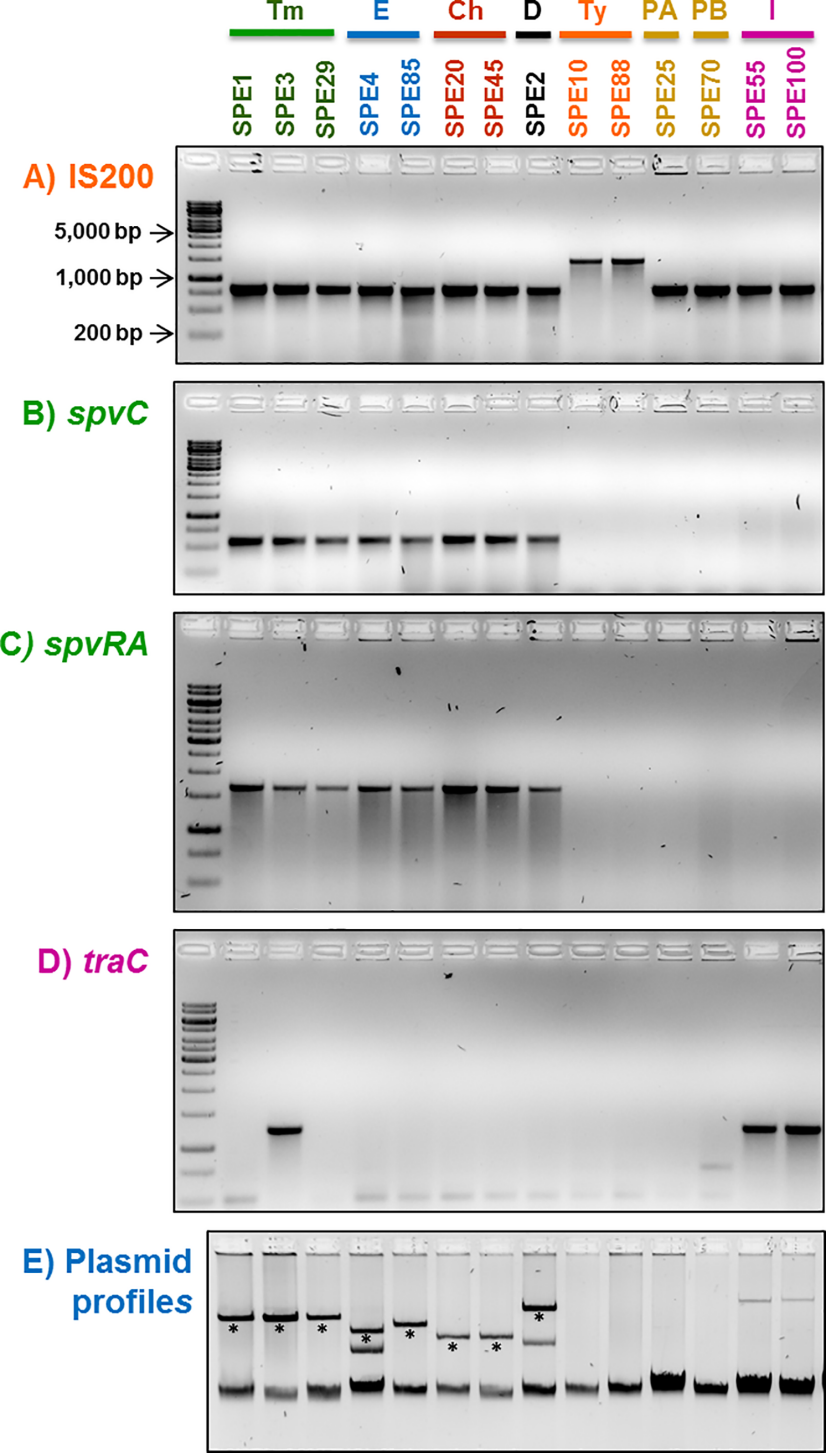
PCR typing of representative blood isolates from Lima hospitals using different gene markers. A) The *gyrA-*IS200-*rcsC* amplification distinguishes serovar Typhi from other serovars. B) and C) The *spvC* and *spvRA* markers detect the virulence plasmid (pSV). D) *traC* is a marker for the megaplasmid of Infantis (pESI). E) Plasmid profiles of the same strains. The asterisks below the bands in the plasmid profiles indicate the plasmid suspected to be the pSV. Tm, Typhimurium; E, Enteritidis; Ch, Choleraesuis; Ty, Typhi; PA, Paratyphi A; PB, Paratyphi B; and I, Infantis.

The Typhi isolates were also confirmed by amplification of the *gyrA-*IS200-*rcsC* region. Using specific primers this region renders a band of about 1.5 kb for Typhi strains, and a band of 0.8 kb for strains belonging to other *Salmonella* serovars, allowing the rapid differentiation of Typhi from other serovars (Fig 1A).

The classical serotyping agglutination method was applied to 30 selected isolates, including four and eleven identified as Enteritidis or Typhimurium, respectively, according to the multiplex PCR typing method (Table 1). Consistently, these isolates were confirmed to be Enteritidis and Typhimurium (Table 1). Further, five out of the six isolates that could not be assigned to a serovar by PCR typing, were successfully typed by serology as Choleraesuis (n = 3), Paratyphi A (n = 1) and Dublin (n = 1). Isolate SPE45 could not be classified either by the serological approach (Table 1). In addition, isolate SPE70, classified as Paratyphi B by the multiplex PCR, could not be confirmed by the serological method (Table 1).

Taken altogether, our results showed that PCR typing methods consistently allowed assigning the serovar to the vast majority (94%) of the isolates, and that classical serotyping could resolve most others. Of note, isolate SPE45 could not be classified by any of these methods.

### The *hemD* and *purE* genes of the MLST scheme contain enough discriminating typing information to classify most *Salmonella* blood isolates

Genotyping of bacterial isolates has been widely achieved by using the nucleotide sequence-based approach known as MLST. For *S*. *enterica*, MLST relies on the comparison of internal sequences of seven housekeeping genes (*aroC*, *dnaN*, *hemD*, *hisD*, *purE*, *sucA* and *thrA*) [4]; however, the procedure is time consuming and expensive. In order to implement a more practical and economic screening of the Peruvian isolates, we inspected the EnteroBase (http://enterobase.warwick.ac.uk/species/index/senterica) in search of loci with high variability and discriminatory power among *Salmonella* serovars. Based on this analysis, we found that the *hemD* and *purE* sequences, from the seven-loci MLST scheme [34], discriminate among the most prevalent Enteritidis and Typhimurium STs, respectively, and also discriminate within the most common sequence types (STs) of these serovars, thus an initial two-loci analysis of the complete collection of blood isolates was performed by amplifying and sequencing the partial sequence of the *hemD* and *purE* genes (Table 1). The resulting sequences were introduced into the *Salmonella* MLST database for allele number assignment (http://mlst.warwick.ac.uk/mlst/dbs/Senterica).The *hemD* and *purE* allele combination was unequivocal to reveal the serovar adscription of isolates of serovars Typhimurium, Typhi, Paratyphi A, Paratyphi B and Infantis (highlighted in boldface in Table 1), when compared with the complete MLST database at the EnteroBase. For the Enteritidis, Dublin and Choleraesuis isolates, the distinct *hemD* and *purE* combinations left open the possibility for more than one serovar. For example, the *hemD3*-*purE6* combination was found in 15,367 isolates reported in the EnteroBase (April 2017), among them 15,156 belonged to serovar Enteritidis, 108 to Paratyphi B monophasic and 65 to serovar Concord. On the other hand, the *hemD35-purE26* combination suggested that isolate SPE45 belonged to serovar Choleraesuis. Of the isolates reported in the EnteroBase with this combination of alleles, 60% correspond to serovar Choleraesuis var. Kunzendorf, while among the other 40% there are several *Salmonella* isolates belonging to serovars Typhisuis or Paratyphi C. Further confirmation of SPE45, SPE68, SPE20 and SPE34 as Choleraesuis var. Kunzendorf came from the complete seven-loci MLST (see below).

The seven-loci MLST scheme was applied for a selection of the isolates in order to validate the results obtained by the two-loci scheme. In addition to *hemD* and *purE*, the partial sequences for the remaining five loci (*aroC*, *dnaN*, *hisD*, *sucA* and *thrA*) were obtained for 18 representative isolates of the serovars present in the *Salmonella* collection (Table 1). In all cases the data from the seven-loci confirmed the serovar assignment obtained by the *hemD-purE* two-loci scheme. On the other hand, ST assignment showed that all isolates of the same serovar belonged to the same ST (Table 1 and Table 2). The STs found were 11, 19, 10, 85 and 32 for Enteritidis, Typhimurium, Dublin, Paratyphi A and Infantis, respectively, which are the most abundant genotypes for these serovars in EnteroBase, indicating that these blood isolates correspond to the most abundant genotypes worldwide.

**Table 2:** Multilocus sequence typing of 18 selected *Salmonella* isolates from Lima, Peru*.

Strain	Year	*aroC*	*dnaN*	*hemD*	*hisD*	*purE*	*sucA*	*thrA*	ST	Predicted serovar
SPE78	2008	*5*	*2*	*3*	*7*	*6*	*6*	*11*	11	Enteritidis
SPE85	2008	*5*	*2*	*3*	*7*	*6*	*6*	*11*	11	Enteritidis
SPE4	2009	*5*	*2*	*3*	*7*	*6*	*6*	*11*	11	Enteritidis
SPE32	2010	*5*	*2*	*3*	*7*	*6*	*6*	*11*	11	Enteritidis
SPE7	2008	*10*	*7*	*12*	*9*	*5*	*9*	*2*	19	Typhimurium
SPE3	2009	*10*	*7*	*12*	*9*	*5*	*9*	*2*	19	Typhimurium
SPE87	2009	*10*	*7*	*12*	*9*	*5*	*9*	*2*	19	Typhimurium
SPE1	2010	*10*	*7*	*12*	*9*	*5*	*9*	*2*	19	Typhimurium
SPE45	2008	*36*	*31*	*35*	*14*	*26*	*34*	*8*	68	Choleraesuis var Kunzendorf
SPE20	2011	*36*	*31*	*35*	*14*	*26*	*34*	*8*	68	Choleraesuis var Kunzendorf
SPE34	2011	*36*	*31*	*35*	*14*	*26*	*34*	*8*	68	Choleraesuis var Kunzendorf
SPE95	2008	*1*	*1*	*2*	*1*	*1*	*1*	*5*	2	Typhi
SPE88	2010	*1*	*1*	*2*	*1*	*1*	*1*	*5*	2	Typhi
SPE25	2010	*45*	*4*	*8*	*44*	*27*	*9*	*8*	85	Paratyphi A
SPE70	2008	*2*	*14*	*24*	*14*	*37*	*19*	*8*	86	Paratyphi B
SPE2	2009	*5*	*2*	*3*	*6*	*5*	*5*	*10*	10	Dublin
SPE55	2011	*17*	*18*	*22*	*17*	*5*	*21*	*19*	32	Infantis
SPE100	2013	*17*	*18*	*22*	*17*	*5*	*21*	*19*	32	Infantis

* Allele, ST and predicted serovar assignments were based on the *Salmonella enterica* MLST database (http://mlst.warwick.ac.uk/mlst/dbs/Senterica/).

These results confirm the practicality and reproducibility of the MLST data as a powerful and feasible *Salmonella* typing method, and support the widely accepted proposal to replace classical serotyping by MLST [4]. The main benefit of MLST data is that it allows comparison of newly generated information anywhere in the world with an extensive database, EnteroBase (http://enterobase.warwick.ac.uk/species/index/senterica), which contains the ST for over 84,400 *Salmonella* strains (April 2017). In addition, our results also show that a two-loci scheme can also provide useful information for a large number of isolates.

### *Salmonella* blood isolates displayed low levels of antimicrobial resistance

As for many bacterial infectious diseases, antibiotic resistance in *Salmonella* has increased over the years becoming a major health concern around the world [41]. Increasing resistance to multiple antibiotics is frequently associated to the acquisition of multiple drug resistance gene cassettes carried on integrons [28]. Specific primers corresponding to conserved sequences (S1 Table) were used to probe by PCR for the presence of integrons among the *Salmonella* isolates. Most of the isolates did not carry integrons and only three of them were positive for the amplification of integron gene-cassettes. Infantis isolates SPE55 and SPE100 displayed an amplification band of about 1,000 bp, whose sequence revealed to be an *aadA1* gene, coding for streptomycin and spectinomycin resistance. Dublin isolate SPE2 carried a 2,000 bp integron coding for *dfrA1* and *aadA1* genes, conferring resistance to trimethoprim, streptomycin and spectinomycin.

In addition, all 95 blood-culture isolates were tested for susceptibility to ten primarily used antimicrobial drugs. Most of the isolates (74%) were not resistant (NR) to all the tested antibiotics, and only three, SPE55 and SPE100 (Infantis) and SPE39 (Enteritidis), were resistant to more than two drugs, therefore considered as multidrug-resistant (MDR) (Table 1). The most frequently found resistance was to nalidixic acid, detected in 14 of the 95 isolates. It is worth mentioning that, although only one isolate was resistant to ciprofloxacin, 35 isolates showed intermediate susceptibility to this quinolone. These include 26 NR isolates and 9 isolates resistant to nalidixic acid (Table 1).

### Most of the blood-isolates harbor plasmids

Large, low-copy-number plasmids carrying virulence or antibiotic resistance genes are a common trait of particular *Salmonella* serovars. To determine the presence of the virulence plasmid pSV, two specific markers, *spvC* and *spvRA*, were used. PCR amplification was positive in all the isolates of the serovars known to naturally harbor pSV, namely Enteritidis, Typhimurium, Choleraesuis and Dublin (Table 1 and Fig 1B and 1C). In addition, plasmid profiles were generated for representative strains of the eight serovars found in this study. Most of the strains showed the presence of one or two large plasmids (>20 kb), with the exception of all Typhi, Paratyphi A and Paratyphi B isolates, which did not contain visible plasmids. In the Enteritidis, Typhimurium, Choleraesuis and Dublin isolates one of those plasmids corresponded to the approximate size reported for pSV in each serovar (Fig 1E and S3 Fig). Dublin isolate SPE2 showed a large plasmid of about 100 kb, which likely corresponds to a pSV with accessory regions, and could harbor the 2,000 bp integron.

Serovar Infantis isolates SPE55 and SPE100 displayed large plasmids (more than 200 kb) (Fig 1E), which could correspond to the MDR-related megaplasmid pESI (280 kb) reported for other Infantis strains [42, 43]. For this reason, we included in our PCR typing scheme the primers reported by Aviv *et al*. (2016) for the amplification of the *traC* gene, considered part of the pESI plasmid backbone [38]. Both Infantis isolates SPE55 and SPE100 were negative for *spvC* or *spvRA*, indicating that they are not pSV plasmids (Fig 1B and 1C and Table 1). Instead, these plasmids seem to be related to pESI plasmid based on the amplification of *traC* (Fig 1D), and the presence of the 1,000 bp integron (*aadA1*), which could be part of the Tn21 transposon reported in pESI [38]. Unexpectedly, Typhimurium SPE3 was also positive for *traC* (Fig 1D). SPE3 displayed a single large plasmid of the size of the Typhimurium pSV (about 95 kb) and was positive for *spvC* and *spvRA*, which supports that this isolate carries a pSV. The basis for the positive *traC* result needs further studies.

### RAPD fingerprints discriminate between serovars but not within serovars

Genetic variability within isolates of the predominant serovars was assessed by the RAPD methodology [12]. A preliminary screening using primers OPB-15, OPB-17 and P1254 was performed for a sample of 20 representative isolates from different hospitals and years of isolation. Only primer OPB-15 produced polymorphic banding patterns and therefore used to generate RAPD fingerprints for all the 95 isolates. Low fingerprint variability was detected within serovars. The most variable serovar was Typhimurium for which three different banding patterns were detected, with 27 isolates with pattern B, four with C and three with D. All the 42 Enteritidis isolates displayed the same RAPD pattern A, the three isolates classified as Choleraesuis displayed pattern G, and only one Typhi isolate displayed a distinct pattern (F) compared to the E pattern displayed by the other nine Typhi isolates (Table 1). Despite the low variability within serovars, the RAPD fingerprints clearly distinguished isolates from different serovars (S4 Fig). Importantly, it provided additional evidence for the assignment of the undetermined SPE45 isolate to Choleraesuis since it displayed the G pattern (Table 1).

## Discussion

This is the first report analyzing isolates causing bacteremia in Lima, which provides relevant results to understand the epidemiology of invasive *Salmonella* in Peru and to highlight the importance of establishing molecular typing methods in the region for a timely assessment of the strains causing invasive salmonellosis. Isolates of *Salmonella enterica* causing bacteremia in patients from nine public hospitals in Lima, Peru, between 2008 and 2013, were typified at serovar level by an approach involving several molecular typing tools. Our results are summarized as follows: 1) The multiplex PCR along with the two-loci sequencing scheme allowed to determine the serovar for all the isolates; 2) Eight serovars were the cause of invasive disease in Lima during this period, the most abundant serovars were Enteritidis, Typhimurium and Typhi; 3) The isolates displayed low levels of antimicrobial resistance to 10 antibiotics, with only three MDR isolates, and three isolates carried resistance integrons, including one Dublin isolate and the two serovar Infantis isolates, which harbored large plasmids; 4) A large proportion of the isolates (37%) showed intermediate susceptibility to ciprofloxacin (ISC); 5) The isolates of serovars known to carry the virulence plasmid pSV (Enteritidis, Typhimurium, Choleraesuis and Dublin) harbored this genetic element.

The prevalence of *Salmonella* serovars isolated from invasive cases of salmonellosis varies depending on the world region. Typhimurium and Enteritidis are the two most frequently reported serovars, as they are responsible for most human salmonellosis cases all around the world; however, the incidence of other serovars such as Infantis seems to be on the raise [44]. Our results show that the serovars associated to invasive infection in Lima are similar to those described in other regions.

The Enteritidis isolates were highly homogeneous, displaying the same RAPD fingerprint pattern, all harbored pSV, and the three MLST sequenced isolates were ST11. This is considered the founder genotype for the Enteritidis population, and has been reported in other countries of South America [45, 46]. Only one Enteritidis isolate was MDR, but no evidence of integrons was found, and 16 had ISC.

The Typhimurium isolates showed more variability than Enteritidis, displaying three RAPD patterns (B, C and D). However, MLST sequencing of isolates showing these RAPD patterns (SPE1 [B], SPE3 [C], SPE7 [B] and SPE87 [D]) were ST19 (Table 2), which is found worldwide, including other South American countries (http://enterobase.warwick.ac.uk/species/index/senterica) and Mexico [29, 44]. All the Typhimurium isolates harbored pSV, most were not resistant to the antibiotics tested, few displayed ISC, and none carried integrons. This is in contrast with the results found for Typhimurium in Mexico, which displayed higher diversity with four STs (ST19, ST213, ST302 and ST429). Moreover, a third of the ST19 Mexican isolates carried the *Salmonella* genomic island inserted into the chromosome, which includes a MDR region with two integrons. However, the Mexican Typhimurium population was isolated from more diverse sources (animal and human), and the systemic infection cases were mainly produced by ST213 isolates, which lacked pSV and harbored MDR IncA/C plasmids [29]. Another Typhimurium genotype that has drawn the attention of the research community is ST313, which has been associated with invasive infections in Africa [47, 48]. ST313 and ST213 are single-locus variants of ST19, differing only in their *purE* alleles; therefore, the two-loci typing scheme used for the Peruvian isolates was designed to detect the presence of these genotypes, yet only ST19 were found.

The third most abundant serovar was Typhi with 10 isolates; however, it is worth mentioning that before starting this study, 33 out of an initial total of 127 blood isolates were determined to be Typhi by biochemical test and thus not included as this study was intended to analyze non-typhoidal *Salmonella*. Taking this into consideration, Typhi was in fact the most abundant serovar in this survey with 43 out of 127 isolates, followed closely by Enteritidis with the 42 isolates. The ten additional Typhi isolates studied here were highly homogeneous, displaying identical RAPD fingerprints (with one exception), the same *hemD* and *purE* sequence alleles (Table 1), and also lacked plasmids (Fig 1D). The MLST sequence of three selected isolates suggested that the Typhi Peruvian strains belong to the abundant founder ST2 genotype. Typhi ST1 and ST2 genotypes differ only in the *hemD* allele; therefore, the two-locus scheme allowed discerning between these genotypes.

Quinolone resistance in *Salmonella* spp. can be associated to mutations in the *gyrA* and *gyrB* genes or in the *parC* and *parE* genes, coding for the DNA gyrase and topoisomerase, respectively [49]. In this regard, the referred 33 Typhi isolates not included in the present work were previously used to screen for mutations in the quinolone resistance-determining region of each gene. All ISC isolates showed a mutation at codons 83 or 87 of *gyrA*, which result in resistance to NAL, or at codon 464 of *gyrB*, which is associated with non-classical quinolone resistance [50]. Five of the ten Typhi isolates reported here showed ISC regardless of their susceptibility to NAL, suggesting that they carry similar *gyrA* or *gyrB* mutations. ISC cannot be predicted by testing for NAL resistance as routinely done, thus posing the risk of therapeutic failure [51]. Taking this into consideration, it would be interesting to determine the genetic basis of the ISC isolates of other serovars susceptible to NAL.

The role of the pSV plasmid in causing systemic infections in humans remains controversial, as strains of serovars Typhimurium and Enteritidis lacking this plasmid have been reported from invasive cases of human salmonellosis [18, 22, 23, 29, 50, 52]. Despite these observations, the pSV plasmid was detected in all the isolates causing bacteremia in Peru of the serovars known to harbor it, supporting the notion that this plasmid has a role in the production of the systemic infections.

Since *Salmonella* is a major cause of infection worldwide, it is necessary to determine the identity of the serovar as efficiently and timely as possible, in order to setup preventive and control epidemiological actions. The classical serological analysis is labor intensive, time consuming and costly. In this report we show that the use of a combination of molecular tools to address the serovar identity of a collection of diverse isolates was highly effective to unequivocally assign their serovar to all of them. Substitution of serotyping by molecular methods is considered a more efficient and cost-effective alternative, as previously suggested by other authors [53, 54].

The multiplex PCRs designed to discriminate among commonly found serovars [7, 13] could be routinely used to provide an economic and robust way towards a first determination of the serovar (S5 Fig). If serovar Typhi is suspected, a rapid confirmatory screening can be performed using the *gyrA-*IS200-*rscC* PCR, which easily discriminates between Typhi and other serovars [14, 15]. The two-loci sequence typing was also very efficient in classifying most of the serovars present in the strain collection reported here. When the serovar of an isolate is suspected and there is an exclusive allele for that serovar (highlighted in boldface in Table 1), for example, *hemD22* is exclusive for Infantis, and sequencing of one locus can be used to confirm the finding. In fact, there is an increase in the number of Infantis infections in Peru, now being the third most frequently isolated *Salmonella* serovar [30], making this marker a useful tool to trace Infantis infections.

If there is an interest to place into the worldwide context an isolate, the complete seven-loci MLST scheme is recommended (S5 Fig). This provides a multilocus genotype (ST), which has the possibility of being compared with thousands of isolates reported from several countries around the world. If known alleles or ST are found, the isolates can be submitted to the MLST website, making them available to the public [4]. The drawback of the MLST method is that the databases (MLST and EnteroBase) nowadays accept only the description of new alleles or STs based on the short-reads generated by complete genome sequencing of the strains, which is unaffordable for the routine laboratory processing of isolates in developing countries. Nevertheless, molecular epidemiology is moving towards sequencing of complete genomes [55–57], and for particular isolates this alternative would provide the raw material to perform analysis that can be used to address a wide range of issues, from antimicrobial therapy to evolutionary analyses.

## Supporting information

S1 Text Detailed protocols for DNA and plasmid extraction procedures. (PDF)

S1 Table Primers used in this study. (PDF)

S1 Fig Geographic distribution and number of *Salmonella* isolates per hospital during 2008 to 2013 in Lima, Peru. (TIFF)

S2 Fig Multiplex PCR serotyping. (TIFF)

S3 Fig Plasmid profiles of representative strains. (TIFF)

S4 Fig Representative RAPD fingerprint profiles for the nine *Salmonella* serovars found in Lima, Peru. (TIFF)

S5 Fig Flowchart showing the typing scheme proposed in this work. (TIFF)
