# Supplementary material for: Characterization of Salmonella enterica isolates causing bacteremia in Lima, Peru, using multiple typing methods
Source: PLoS One. 2017 Dec 21;12(12):e0189946. doi: 10.1371/journal.pone.0189946 (PMC5739443; doi:10.1371/journal.pone.0189946)
Supplement: S1 Text — (PDF) [file pone.0189946.s001.pdf]

**Characterization of *Salmonella enterica* isolates causing bacteremia in Lima, Peru, using multiple typing methods. Silva *et al*. PLOS ONE (2017).**

**S1 Text. Detailed protocols for DNA and plasmid extraction procedures.**

**A) DNA extraction by boiling (modified from Lin *et al*. 1996).**

1) Take one colony from an LB petri dish culture and resuspend in 150 ul of miliQ water in a 1.5 ml plastic tube.

2) Centrifuge at 13,000 rpm for 3 mins. Discard the supernatant and resuspend the pellet in 150 ul of miliQ water.

3) Make a hole in the tube cap with a needle. Incubate the tubes in boiling water or in a thermomixer for 5 minutes at 95$^{o}$C. Cool down the tubes at 4$^{o}$C for 5 minutes.

4) Centrifuge at 13,000 rpm for 5 mins and transfer the supernatant to a clean 1.5 ml plastic tube. The lysate is ready to use as DNA template or store at -20$^{o}$C until use.

**B) DNA extraction from liquid cultures by the salting out procedure (modified from Miller *et al*. 1988).**

1) Take 1.5 ml from a bacterial overnight LB culture and harvest by centrifugation at 12,000 rpm for 2 mins. Discard the supernatant and resuspend the pellet in 0.5 ml of buffer SET (50 mM Tris-HCl, pH 8.0, 50 mM EDTA, 20% w/v Sucrose).
2) Add 30 μl of 20% SDS and vortex briefly. Incubate at 80ºC for 5 mins and then cool down the tubes at 4$^{o}$C for 5 minutes. Add 5 μl of RNase (10mg/ml), mix gently and incubate at 37ºC for 50 mins. Cool down the tubes at 4$^{o}$C for 5 minutes.
3) Add 200 μl of 5M NaCl, vortex and incubate on ice for 15 mins. Centrifuge at 13,000 rpm for 10 mins and transfer the supernatant to a clean 1.5 ml plastic tube.
4) Add 0.7 ml of isopropanol and centrifuge at 13,000 rpm for 15 mins. Discard the supernatant, add 0.8 ml of 70% ethanol and centrifuge at 13,000 rpm for 5 mins. Repeat twice.
5) Discard the supernatant, let the tubes drip the ethanol drops and air-dry for 15 mins. Suspend the DNA pellet in 120 μl of 10 mM TE buffer (Tris 10 mM, EDTA 1mM).

**C) Plasmid extraction alkaline lysis procedure (modified from Kieser 1984)**

1) Harvest 3 ml of an overnight LB culture by centrifugation at 10,000 rpm for 2 mins. Discard the supernatant and suspend the pellet in 0.2 ml of buffer SET (50 mM Tris-HCl, pH 8.0, 50 mM EDTA, 20% w/v Sucrose).
2) Add 50 ul of lysozyme (10mg/ml), gently mix and incubate on ice for 30 mins. Add 100 µl of lysis solution (0.3 M NaOH, 2% SDS), gently mix and incubate at 55$^{o}$C for 30 mins. Cool down the tubes at 4$^{o}$C for 5 minutes.
3) Add 25 µl of phenol and 25 µl of chloroform-isoamyl alcohol (24:1), vortex and centrifuge at 13,000 rpm for 20 mins at 4$^{o}$C.
4) Transfer 250 µl of the supernatant to a clean 1.5 ml plastic tube. The plasmid preparation is ready to use or store at -20$^{o}$C until use.

**References.**

Lin, A.W., Usera, M.A., Barrett, T.J., and Goldsby, R.A. *Application of random amplified polymorphic DNA analysis to differentiate strains of Salmonella enteritidis.* J. Clin. Microbiol., 1996. 34(4): p. 870-6.

Miller, S.A., Dykes, D.D., and Polesky, H.F. *A simple salting out procedure for extracting DNA from human nucleated cells.* Nucleic Acids Res., 1988. 16(3): p. 1215.

Kieser, T. *Factors affecting the isolation of CCC DNA from Streptomyces lividans and Escherichia coli.* Plasmid, 1984. 12(1): p. 19-36.
