# Supplementary material for: Characterization of Salmonella enterica isolates causing bacteremia in Lima, Peru, using multiple typing methods
Source: PLoS One. 2017 Dec 21;12(12):e0189946. doi: 10.1371/journal.pone.0189946 (PMC5739443; doi:10.1371/journal.pone.0189946)
Supplement: S1 Table — (PDF) [file pone.0189946.s002.pdf]

Table S1. Primers used in this study.

Name	Gene	Sequence	Size bp	T °C[a]	Ref.[b]
**Serogroup**					(1)
F-tyvD	*tyvD*	GAGGAAGGGAAATGAAGCTTTT	614 pb	58	
R-tyvD	*tyvD*	TAGCAAACTGTCTCCCACCATAC			
F-wzxB	*wzx*	GGCATATATTTCTGTATTCGC	229 pb	58	
R-wzxB	*wzx*	GCCTTAATTAAGTAAGTTAGTGGAAGC			
F-wzxC1	*wzx*	CAGTAGTCCGTAAAATACAGGGTGG	483 pb	58	
R-wzxC1-mod	*wzx*	AATGCTATAAATACTGTGTTAAATTGC			
F-wzxC2	*wzx*	ACTGAAGGTGGTATTTCATGGG	153 pb	58	
R-wzxC2	*wzx*	AAGACATCCCTAACTGCCCTGC			
F-wzxE1	*wzx*	TAAAGTATATGGTGCTGATTTAACC	344 pb	58	
R-wzxE1	*wzx*	GTTAAAATGACAGATTGAGCAGAG			
**H1 flagella**					(2)
Sense-60*	*fliC*	GCAGATCAACTCTCAGACCCTGGG		58	
Antisense-i	*fliC*	ATAGCCATCTTTACCAGTTCC	250 pb		
Antisense-z_{10}	*fliC*	CGTCGCAGCTTCTGCAACC	447 pb		
Antisense-b	*fliC*	CGCACCAGTCYWACCTAAGGCGG	167 pb		
Antisense-eh	*fliC*	AACGAAAGCGTAGCAGACAAG	200 pb		
Antisense-lv	*fliC*	CCTGTCACTTTCGTGGTTAT	326 pb		
Antisense-r	*fliC*	AAGTGACTTTTCCATCGGCTG	281 pb		
Forward-d	*fliC*	CCCGAAAGAAACTGCTGTAACCG	100 pb	58	
Reverse-d	*fliC*	TGGATATCAGTATTGCTCTGGGC			
Forward-G	*fliC*	GTGATCTGAAATCCAGCTTCAAG	500 pb	58	
Reverse-G	*fliC*	AAGTTTCGCACTCTCGTTTTTGG			
Forward-Sdf-l	*sdf1*	TGTGTTTTATCTGATGCAAGAGG	293 pb	58	
Reverse-Sdf-l	sdf1	CGTTCTTCTGGTACTTACGATGAC			
**H2 flagella**					(3)
Sense-F1mod**	*fljB*	CTTATGCCRATAATGGTACTACACTG		58	
Antisense-R5mod	*fljB*	GGTTACAGVAGCCGTACCAG	98		
Antisense-R6	*fljB*	CTCCTGTACTTCTGTTTTGGTTGTA	298		
Antisense-R7	*fljB*	TAATCGCCATTTTTGTCGAG	190		
Antisense-R1mod	*fljB*	TTGACCAAYKYMGCGSCAT	388		
Sense-Fw	*fljB*	GTGGGGCAACMCTCAATACTG	240	58	
Antisense-Rw	*fljB*	CCTGCCACTTTCGTGGTTGC			
Sense-Fe***	*fljB*	GGCAACCCGACAGTAACTGGCGATAC			

Antisense-Rx	*fljB*	CCATCCTTAAAGGATACGGC	54	58	
Antisense-Rz$_{15}$	*fljB*	ATCAACGGTAACTTCATATTTG	134		
**RAPD**					(4)
OPB-15		GGAGGGTGTT	Variable	35	
OPB-17		AGGGAACGAG	Variable	35	
P1254		CCGCAGCCAA	Variable	35	
**MLST**					(5)
aroC	*aroC*	CCTGGCACCTCGCGCTATAC<br>CCACACACGGATCGTGGCG	826 pb	55	
dnaN	*dnaN*	ATGAAATTTACCGTTGAACGTGA<br>AATTTCTCATTCGAGAGGATTGC	833 pb	55	
hemD	*hemD*	GAAGCGTTAGTGAGCCGTCTGCG<br>ATCAGCGACCTTAATATCTTGCCA	666 pb	55	
hisD	*hisD*	GAAACGTTCCATTCCGCGCAGAC<br>CTGAACGGTCATCCGTTTCTG	894 pb	55	
purE	*purE*	ATGTCTTCCCGCAATAATCC<br>TCATAGCGTCCCCCGCGGATC	510 pb	55	
sucA	*sucA*	AGCACCGAAGAGAAACGCTG<br>GGTTGTTGATAACGATACGTAC	643 pb	55	
thrA	*thrA*	GTCACGGTGATCGATCCGGT<br>CACGATATTGATATTAGCCCG	852 pb	55	
**PCR typing**					
IS200A5	*gyrA*-IS200-*rcsC*	GGTGCGTACCCGAGTGTC	Variable	55	(6)
IS200B3		CTGCCAATCAGGAAAACGCG			
spvC-1	*spvC*	ACTCCTTGCACAACCAAATGCGGA	550	55	(7)
spvC-2		TGTCTCTGCATTTCGCCATCA			
spvRA-F	*spvAR*	ATGGCATCATTAACCACCAT	1,676	55	This study
spvRA-R		TGAGCAGGGTTATTTCAGAC			
traC Fw		CCAGTATTTCCCGGCCTTCG		55	(8)
traC Rv		GTTATCCGGCGTGCAGAAAC			
CS-F	Casette	GGCATCCAAGCAGCAAG	Variable	55	(9)
CS-R		AAGCAGACTTGACCTGA			

[a] Annealing temperature used in the PCR amplification cycles.

[b] Source reference.

*Sense 60 is used as forward primer for antisense-i -lv -b -z10 -eh and -r

** Sense F1mod is used as forward primer for antisense-R1 (to detect antigen 1,2) -R5 (to detect antigen 1,5) -R6 (to detect antigen 1,6) and R7 (to detect antigen 1,7)

***Sense Fe is used as forward primer for antisense Rx (to detect antigen e,n,x) and Rz15 (to detect antigen e,n,z15)

References.

1. Herrera-Leon S, Ramiro R, Arroyo M, Diez R, Usera MA, Echeita MA. 2007. Blind comparison of traditional serotyping with three multiplex PCRs for the identification of *Salmonella* serotypes. Res Microbiol 158:122-7.
2. Echeita MA, Herrera S, Usera MA. 2001. Atypical, fljB-negative *Salmonella enterica* subsp. *enterica* strain of serovar 4,5,12:i:- appears to be a monophasic variant of serovar Typhimurium. J Clin Microbiol 39:2981-3.
3. Herrera-Leon S, McQuiston JR, Usera MA, Fields PI, Garaizar J, Echeita MA. 2004. Multiplex PCR for distinguishing the most common phase-1 flagellar antigens of Salmonella spp. J Clin Microbiol 42:2581-6.
4. Lin AW, Usera MA, Barrett TJ, Goldsby RA. 1996. Application of random amplified polymorphic DNA analysis to differentiate strains of *Salmonella enteritidis*. J Clin Microbiol 34:870-6.
5. Kidgell C, Reichard U, Wain J, Linz B, Torpdahl M, Dougan G, Achtman M. 2002. *Salmonella typhi*, the causative agent of typhoid fever, is approximately 50,000 years old. Infect Genet Evol 2:39-45.
6. Martinez-Gamboa A, Silva C, Fernandez-Mora M, Wiesner M, Ponce de Leon A, Calva E. 2015. IS200 and multilocus sequence typing for the identification of *Salmonella enterica* serovar Typhi strains from Indonesia. Int Microbiol 18:99-104.
7. Chiu CH, Ou JT. 1996. Rapid identification of *Salmonella* serovars in feces by specific detection of virulence genes, *invA* and *spvC*, by an enrichment broth culture-multiplex PCR combination assay. J Clin Microbiol 34:2619-2622.
8. Aviv G, Rahav G, Gal-Mor O. 2016. Horizontal Transfer of the *Salmonella enterica* Serovar Infantis Resistance and Virulence Plasmid pESI to the Gut Microbiota of Warm-Blooded Hosts. MBio 7.
9. Levesque C, Piche L, Larose C, Roy PH. 1995. PCR mapping of integrons reveals several novel combinations of resistance genes. Antimicrob Agents Chemother 39:185-191.
