# Supplementary figures and images for: Characterization of Salmonella enterica isolates causing bacteremia in Lima, Peru, using multiple typing methods
Source: PLoS One. 2017 Dec 21;12(12):e0189946. doi: 10.1371/journal.pone.0189946 (PMC5739443; doi:10.1371/journal.pone.0189946)

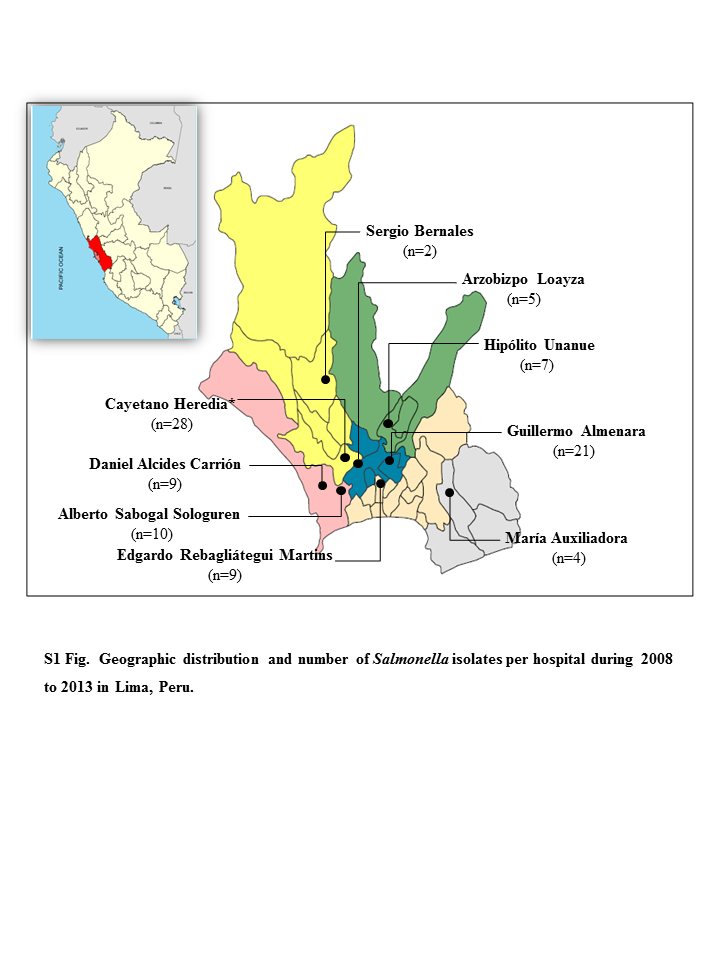

Supplement: S1 Fig — (TIFF) [file pone.0189946.s003.tiff]

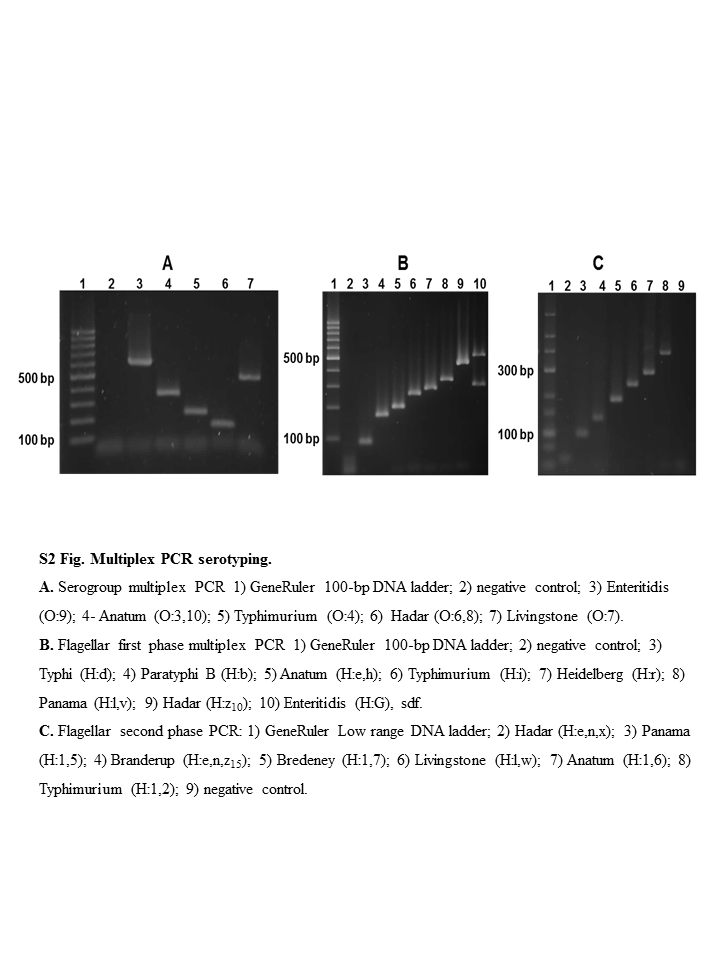

Supplement: S2 Fig — (TIFF) [file pone.0189946.s004.tiff]

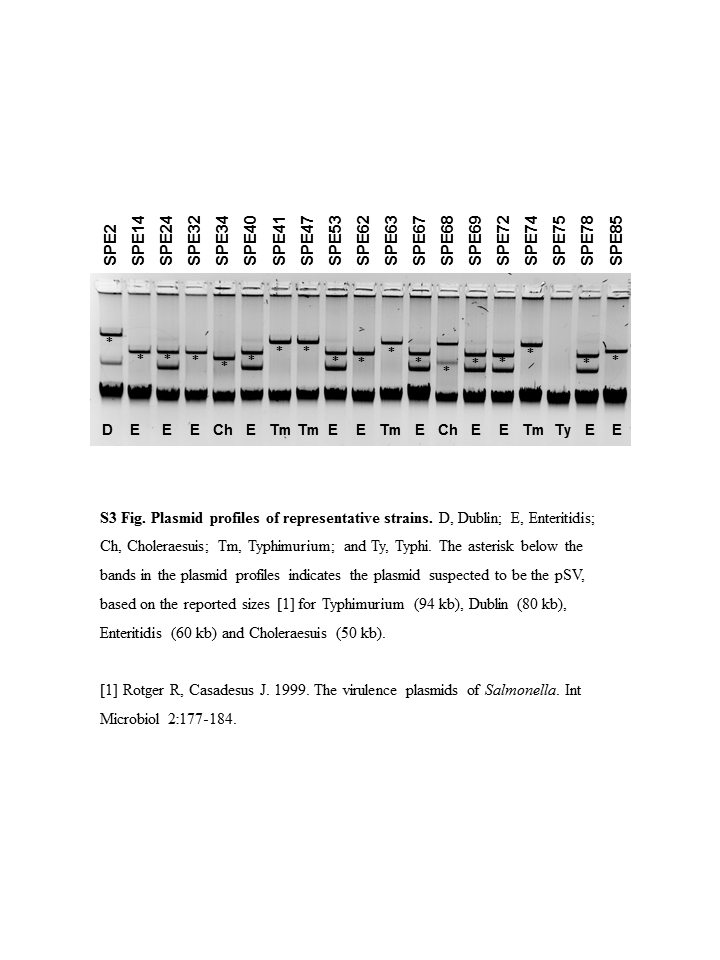

Supplement: S3 Fig — (TIFF) [file pone.0189946.s005.tiff]

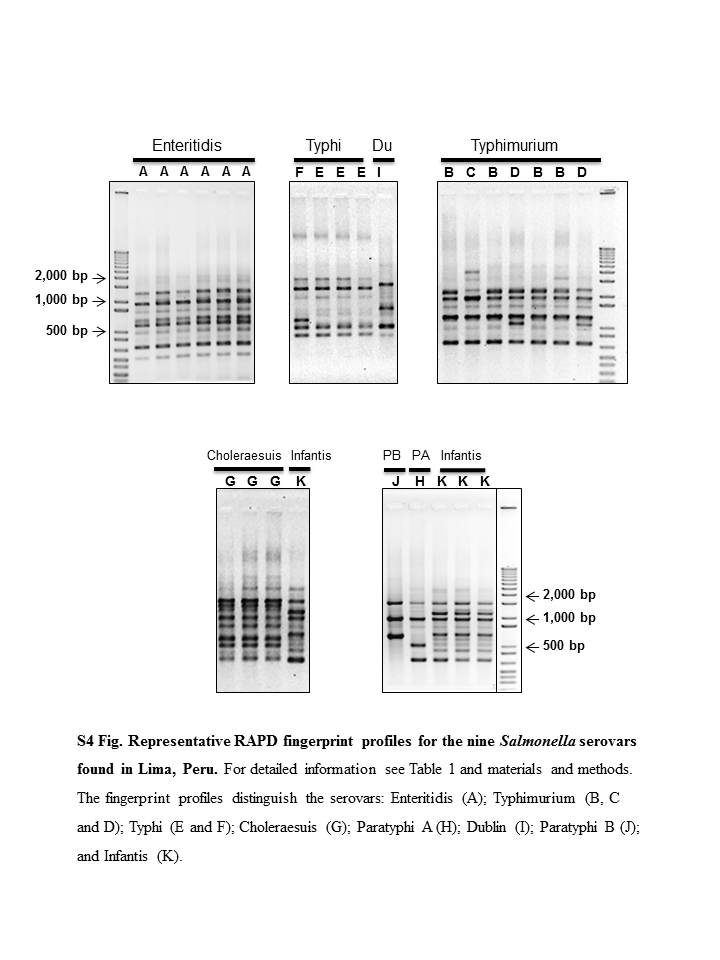

Supplement: S4 Fig — (TIFF) [file pone.0189946.s006.tiff]

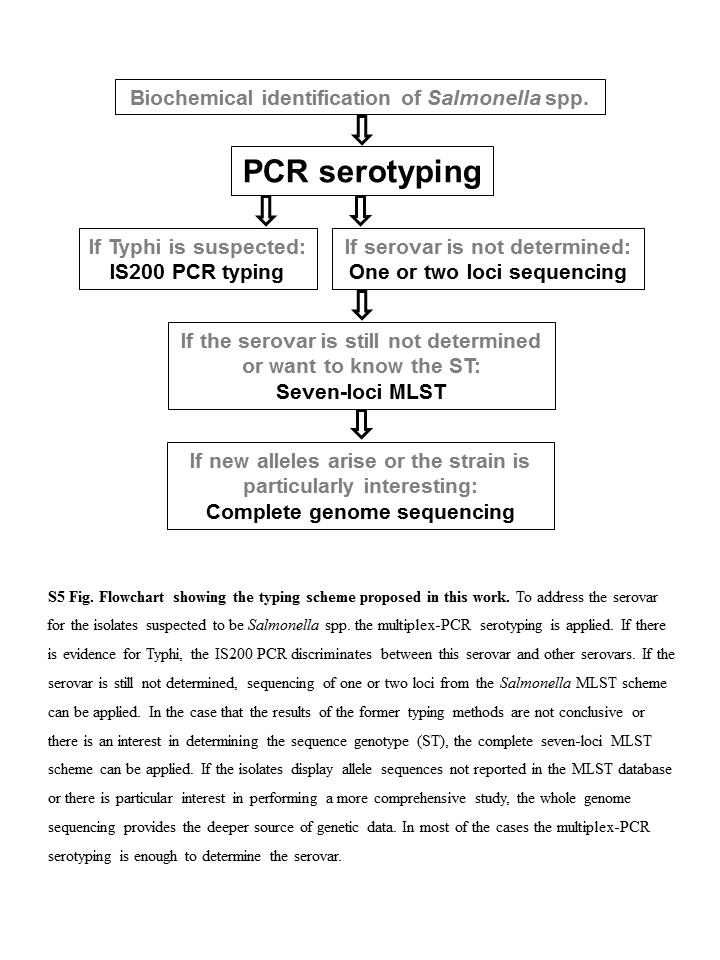

Supplement: S5 Fig — (TIFF) [file pone.0189946.s007.tiff]
